# Can Touchscreen Devices be Used to Facilitate Young Children's Learning? A Meta-Analysis of Touchscreen Learning Effect

**DOI:** 10.3389/fpsyg.2018.02580

**Published:** 2018-12-18

**Authors:** Heping Xie, Ji Peng, Mengyuan Qin, Xuzhe Huang, Fei Tian, Zongkui Zhou

**Affiliations:** ^1^Key Laboratory of Adolescent Cyberpsychology and Behavior (CCNU), Ministry of Education, Wuhan, China; ^2^School of Psychology, Central China Normal University, Wuhan, China; ^3^Air Force Early Warning Academy, Wuhan, China

**Keywords:** touchscreen, physical experience, learning, young children, early childhood education, meta-analysis

## Abstract

Because of the continuous stream of touchscreen apps that are claimed to be educational and the increasing use of touchscreen devices in early childhood, considerable attention is being paid to the effect of touchscreens on young children's learning. However, the existing empirical findings in young child samples are not consistent. In this meta-analysis we tested the overall effect of touchscreen devices on young children's (0- to 5-year-olds) learning performance, as well as moderators of this effect, based on 36 empirical articles (79 effect sizes) involving 4,206 participants. The overall analysis showed a significant touchscreen learning effect (*d* = 0.46), indicating that young children indeed benefited from touchscreen learning. Interestingly, age, learning material domain, comparison group, and experimental environment significantly moderated the effect of touchscreen devices on young children's learning outcome. These findings shed light on the role of touchscreen-related physical experience in early childhood education.

## Introduction

Since Apple launched iPad in 2010, the whole world has begun to be obsessed with a new kind of technical products–touchscreen devices. The popularization of touchscreen devices has stoked public interest in its potential for early childhood education (Rideout, 2014; Hirsh-Pasek et al., 2015; Apple, 2017). By March 2018, Apple reports that there have been over 180,000 educational applications (“apps”) designed specifically for education (Apple, 2018a). In a 2017 nationwide survey by Common Sense Media in the U.S., 98% children from birth to 8 live in a home with mobile devices, 95% of families with children this age have a smartphone, 78% have a tablet, and 42% of children have their own tablet device; 71% parents report that they have downloaded apps (including educational apps) for their children to use; 67% parents whose children use screen media say it helps their child's learning, and 80% of them at least somewhat agree that they are satisfied with the amount and quality of educational screen media available for their children (Rideout, 2017). In addition, touchscreen devices have been gaining wide acceptance in school settings, which has been a global phenomenon (Beach and Castek, 2015; Haßler et al., 2015; McLean, 2016; Chou et al., 2017). For example, with the rapid growth of mobile touchscreen technologies, BYOD (bring your own device) has become a feasible pedagogical strategy which is aimed at promoting students' active engagement during learning (Nortcliffe and Middleton, 2013). BYOD allows students (including young children) to bring their touchscreens or other devices into classrooms for learning goals (Nelson, 2012; New Media Consortium, 2015; Chou et al., 2017). Research showed that 43% of pre-kindergarten through 12th-grade students use mobile devices (e.g., touchscreens) for classroom activities, and they have been adopted as an innovative approach to support traditional learning and teaching practices (New Media Consortium, 2015). That means many children and teachers are authorized to learn and teach by touching the screens, which is more or less different from traditional non-technology-enhanced classroom settings. To some degree, thus, the prevalent enthusiasm for the application of touchscreen devices to early childhood education is literally playing its role in the process that young students learn as well as teachers teach (Hu and Garimella, 2014; New Media Consortium, 2015; Apple, 2017; Papadakis et al., 2017; Chambers et al., 2018).

Touchscreen-based app developers believe that their apps are able to promote young children's learning performance^1^ (Riconscente, 2013; Schroeder and Kirkorian, 2016; Apple, 2017; Herodotou, 2018b). It says on Apple's official website (Apple, 2018b) that iPad apps can help children “stay focused,” “ignite the creativity in every student,” and “bring their biggest ideas to life;” the power and flexibility of iPad can “transform how students learn about and connect with the world around them…make a history lesson as vivid as the present by restoring ancient artifacts, or even peer inside everyday objects to understand how they're put together.” With tools developed for teaching, iPad apps can make it easy for teachers to gain “valuable insight into each student's progress,” “focus on what's most important—teaching,” and even help teachers “evaluate students' long-term progress as they move toward statewide testing.” After highlighting the worldwide amazing success of iPad usage in education, Apple (2017) summarized that using iPad might have the following advantages: (1) improvement in academic performance; (2) increase in engagement and motivation; (3) rise in cost savings and resource efficiency; and (4) integrated focus on content quality and design. From those mentioned above, it seems that touchscreen apps have the potential to make learning and teaching more powerful, which is seemingly beneficial to the improvement of children's learning performance (Wang et al., 2016).

However, the effects of these so-called “educational” apps on learning outcome remain to be largely untested, especially during the early years after the introduction of iPad (Hirsh-Pasek et al., 2015). Only in recent years has this question been extensively and seriously concerned by scholars. The related empirical work has been published in journals such as Science (e.g., Berkowitz et al., 2015), Psychological Science (e.g., Choi and Kirkorian, 2016), Child Development (e.g., Zimmermann et al., 2017), Frontiers in Psychology (e.g., Tarasuik et al., 2018), Computers and Education (e.g., Walczak and Taylor, 2018), Computers in Human Behavior (e.g., Huber et al., 2016), etc. For the same purpose in previous work, the present study focused on reevaluating the impact of educational touchscreen devices on young children's learning outcome (i.e., whether learning by touching a screen could facilitate young children's learning outcome) by conducting a meta-analysis.

### Objective and Rationale

Consider a learning scenario in which a child plays an educational game on a hand-held device such as an iPad. The touchscreen interface of an iPad affords the possibility of physical interactivity such as touching an object on the screen with a finger by a continuous dragging manipulation or by a discrete tapping manipulation (Dubé and McEwen, 2015). The objective of this meta-analysis is to assess the potential pedagogic value of physical interactivity features of touchscreen devices.

The rationale of this meta-analysis is that an assessment of the overall influence of using touchscreen devices on young children's learning outcome is required before widely introducing touchscreen devices to their learning at home or in preschool. In just a few short years, dozens of studies have been conducted to verify the effect of touchscreen devices with physical interactivity features on young children's learning performance (e.g., Aladé et al., 2016; Huber et al., 2016; Kirkorian et al., 2016; Patchan and Puranik, 2016; Schroeder and Kirkorian, 2016). However, the mixed findings (i.e., some studies find positive effects of touchscreen on learning performance, but others find no or even negative effects, see section Research on Young Children's Touchscreen Learning) in this body of research call into question the robustness of this effect. Thus, it is worthwhile to determine whether touchscreen devices usage can work to improve child learning.

### Research on Young Children's Touchscreen Learning

Viewing from a lifespan perspective of cognition, children's knowledge acquisition is likely to be based on their physical experience (Kontra et al., 2012; Loeffler et al., 2016; Setti and Borghi, 2018). This is to some extent in line with the viewpoint of early developmental psychologists (Piaget, 1952; Held and Hein, 1963). In the field of developmental and cognitive science, the notion that physical action and cognition are linked is actually not a novel concept (Glenberg et al., 2013). For instance, Piaget (1952) proposed that knowledge acquired by children is constructed through their actions and it is these body actions that subserve the creation of mental representations which are of importance to information processing. According to his theory, young children, even infants, construct a comprehension of the physical world through their own actions upon and engagement with the world. A body of subsequent research in young child samples confirmed the crucial impact of such physical experience on cognitive processes (e.g., Adolph and Avolio, 2000; Thelen et al., 2001; Smith, 2005; Hadzigeorgiou et al., 2009; Boncoddo et al., 2010; Becker et al., 2014; Mavilidi et al., 2015; Toumpaniari et al., 2015). Besides, effective learning occurs not only when children physically manipulate the materials (Glenberg et al., 2007), but also when they manipulate them in the form of imagination, as long as they possess enough imagining basis in some way, for example, by teaching children how to imagine during learning (Glenberg et al., 2004; Glenberg, 2011).

Because actions play a vital role in the process of young children's cognitive development (Piaget, 1952), it should be beneficial if a certain (virtual) environment is created to strengthen the link between young children's physical experience and their cognitive processing. Touchscreen devices provide a unique and virtual testbed for the effect of physical manipulation on children's learning (Baccaglini-Frank and Maracci, 2015; Wang et al., 2016). Extending the above idea of learning via physical experience and/or actions, scholars have strongly advocated that learning tools in an educational context should be designed in an embodied way (Abrahamson, 2014, 2015; Abrahamson and Lindgren, 2014). A touchscreen device is one of those embodiment-based tools providing access to learning through physical interaction because it invites a child to physically manipulate the elements (e.g., with a finger) presented on the screen. For example, with the help of an iPad a child can scrutinize an object through rotating or zooming it. These sensorimotor interactions and bodily engagement with the touchscreen learning environment contribute to the construction of children's mental representations as well as their cognitive processes (Wang et al., 2016; Yuill and Martin, 2016; Duijzer et al., 2017). Thus, learning from touchscreens is supposed to be potentially beneficial to student performance (Wang et al., 2016).

A series of empirical research has been conducted to examine whether touchscreen learning leads to a stable improvement of young children's learning outcome; however, this outcome has not yet received consistent support (e.g., Huber et al., 2016; Schroeder and Kirkorian, 2016; Wang et al., 2016; Furman et al., 2018), with some studies showing that touchscreen facilitates their learning performance, but others showing that touchscreen does not or even hinders learning performance (see below for details).

On the one hand, some studies have found that there was some beneficial effect of touchscreen devices on young children learning achievement (McKenna, 2012; Schacter and Jo, 2016; Wang et al., 2016; Papadakis et al., 2018). For example, a pre- and post-test study conducted by Wang et al. (2016) found that after 10 min of exposure to an iPad touchscreen app designed to teach how to tell time, the post-test scores of 5- to 6-year-old children were significantly higher than those at pre-test, supporting their prediction that children could benefit from the touchscreen itself. This positive role of touchscreen-based learning in learning outcome has also been proved in a limited number of studies of younger children (e.g., Patchan and Puranik, 2016) and even toddlers (e.g., Strouse and Ganea, 2017). On the other hand, the educational effect of touchscreens on young children's learning outcome has been questioned in some other studies (e.g., Schroeder and Kirkorian, 2016; Piotrowski and Krcmar, 2017; Zipke, 2017). Quite a few studies indicated that learning from touchscreens did not show superiority over other learning methods, for example, learning with physical objects (Huber et al., 2016), learning by watching on touchscreens (Aladé et al., 2016), or face-to-face paper teaching (Kwok et al., 2016). For instance, Aladé et al. (2016) asked preschool-aged children from 45 to 68 months to play an animal measuring game. Results on transfer performance indicated that participants who played the game through touching a tablet did not outperform their counterparts who viewed a video recorded version of the game that was otherwise identical in content to the interactive game. In addition, several studies even discovered a negative impact of touchscreen learning on child performance (e.g., Parish-Morris et al., 2013). Simply put, the mixed findings bring into question the robustness of touchscreen effect with respect to young children's learning. Fortunately, this question could be addressed through meta-analysis to synthesize and test the data from multiple empirical studies.

The above mixed findings at least indicate that not in all cases touchscreen technology has a positive effect on cognitive processing (Wang et al., 2016, p2). Part of the reason might be that there are some potential moderators of this effect. However, to our knowledge, there has been no call for a search for potential moderators in touchscreen learning research and touchscreen scholars have been primarily concerned with the question of whether touchscreen learning works, thus leading to no sufficient knowledge about when it works. To date, dozens of studies have been conducted to verify the touchscreen learning effect in samples of young children under 6 years old (see Table 1), with different characteristics of participants (e.g., age), learning materials (e.g., learning material domain), and experimental designs (e.g., comparison condition, test media, experimental environment). These sets of characteristics are usually regarded as important potential moderators by researchers in the field of learning and instruction (e.g., Berney and Bétrancourt, 2016; Fiorella and Zhang, 2018; Xie et al., 2018). The present meta-analysis will make an attempt to check whether these characteristics moderate the effect of touchscreen on young children's learning outcome.

**Table 1:** A list of studies included in the meta-analysis.

**Study**	**Sample size**	**Age (months)**	**Learning material domain**	**Comparison group**	**Test media**	**Experimental environment**
Aladé et al., 2016	40	mean = 58.06	STEM	watch on touchscreen	paper	laboratory
	40	mean = 58.06	STEM	baseline	paper	laboratory
Bebell and Pedulla, 2015 Exp.1	129	kindergarteners	non-STEM	baseline	paper	classroom
	266	kindergarteners	non-STEM	traditional classroom teaching	paper	classroom
Choi and Kirkorian, 2016	75	mean = 30.04	non-STEM	watch on touchscreen	paper	classroom
Cubelic and Larwin, 2014	291	kindergarteners	non-STEM	traditional classroom teaching	paper	classroom
	144	kindergarteners	non-STEM	baseline	paper	classroom
Furman et al., 2018	42	5–6 years old	STEM	baseline	oral	classroom
	38	5–6 years old	STEM	traditional classroom teaching	oral	classroom
Herodotou, 2018a	18	5 years old	STEM	baseline	paper	classroom
Huber et al., 2016 Exp.1	21	mean = 61.20	non-STEM	baseline	physical objects	laboratory
	50	mean = 61.20	non-STEM	physical objects	physical objects	laboratory
Huber et al., 2016 Exp.2	18	mean = 64.80	non-STEM	baseline	physical objects	laboratory
Kirkorian et al., 2016	38	mean = 25.50	non-STEM	watch on touchscreen	physical objects	classroom
	40	mean = 29.75	non-STEM	watch on touchscreen	physical objects	classroom
	38	mean = 34.00	non-STEM	watch on touchscreen	physical objects	classroom
Krcmar and Cingel, 2014	70	median = 52.50	non-STEM	paper	oral	other
Kwok et al., 2016	86	mean = 66.93	STEM	paper	paper or touchscreen devices	other
	43	mean = 66.93	STEM	baseline	touchscreen devices	other
Mattoon et al., 2015	24	4–5 years old	STEM	traditional teaching	paper	laboratory
	12	4–5 years old	STEM	baseline	paper	laboratory
McKenna, 2012	18	5–6 years old	non-STEM	traditional classroom teaching	paper	classroom
	18	5–6 years old	STEM	traditional classroom teaching	paper	classroom
Moyer-Packenham et al., 2015	35	3–4 years old	STEM	baseline	touchscreen devices	laboratory
	33	5–6 years old	STEM	baseline	touchscreen devices	laboratory
Neumann, 2018	48	mean = 45.19	non-STEM	traditional classroom teaching	paper	other
	24	mean = 45.68	non-STEM	baseline	paper	other
O'Toole and Kannass, 2018	50	mean = 53.04	non-STEM	paper	paper or touchscreen devices	other
	50	mean = 53.04	non-STEM	paper	oral	other
	50	mean = 53.04	non-STEM	baseline	paper or touchscreen devices	other
Oakley et al., 2018	370	5 years old	non-STEM	traditional classroom teaching	paper	classroom
Outhwaite et al., 2018	389	mean = 60.64	STEM	traditional classroom teaching	paper	classroom
	257	mean = 60.70	STEM	baseline	paper	classroom
Papadakis et al., 2018	256	mean = 62.00	STEM	mouse-based computers	paper	classroom
	231	mean = 62.00	STEM	traditional classroom teaching	paper	classroom
	122	mean = 62.00	STEM	baseline	paper	classroom
Parish-Morris et al., 2013 Exp.2	40	mean = 42.14	non-STEM	paper	paper	laboratory
Patchan and Puranik, 2016	32	mean = 51.90	non-STEM	paper	paper	classroom
Piotrowski and Krcmar, 2017	78	mean = 58.80	non-STEM	watch on touchscreen	paper	other
Rattanasone et al., 2016	60	mean = 48.00	non-STEM	baseline	touchscreen devices	other
Robb, 2010	45	mean = 59.23	non-STEM	watch on touchscreen	paper	laboratory
	47	mean = 59.23	non-STEM	paper	paper	laboratory
	45	mean = 59.23	non-STEM	watch on touchscreen	oral	laboratory
	47	mean = 59.23	non-STEM	paper	oral	laboratory
Russo-Johnson et al., 2017 Exp.2	170	mean = 41.05	non-STEM	watch on touchscreen	touchscreen devices	laboratory
Schacter and Jo, 2016	162	mean = 56.00	STEM	traditional classroom teaching	touchscreen devices	classroom
	123	mean = 56.00	STEM	baseline	touchscreen devices	classroom
Schacter and Jo, 2017	378	mean = 564.60	STEM	traditional classroom teaching	touchscreen devices	classroom
Schroeder and Kirkorian, 2016	44	mean = 50.40	STEM	watch on touchscreen	paper	other
	44	mean = 50.40	STEM	watch on touchscreen	physical objects	other
	9	mean = 50.40	STEM	baseline	paper	other
	9	mean = 50.40	STEM	baseline	physical objects	other
Strouse and Ganea, 2017	102	mean = 21.33	non-STEM	paper	paper	laboratory
	75	mean = 21.33	non-STEM	baseline	paper	laboratory
Tarasuik et al., 2017	25	mean = 67.20	non-STEM	baseline	physical objects	laboratory
	24	mean = 45.48	non-STEM	baseline	physical objects	laboratory
	25	mean = 67.20	non-STEM	physical objects	physical objects	laboratory
	24	mean = 45.48	non-STEM	physical objects	physical objects	laboratory
Teepe et al., 2017	71	mean = 40.06	non-STEM	baseline	paper	other
	44	mean = 39.41	non-STEM	baseline	paper	other
Walter-Laager et al., 2017	64	mean = 27.30	non-STEM	paper	paper	other
	31	mean = 27.30	non-STEM	baseline	paper	other
Wang and Chen, in preparation	42	mean = 70.15	STEM	watch on touchscreen	touchscreen devices	other
	41	mean = 70.15	STEM	watch on touchscreen	physical objects	other
	40	mean = 70.15	STEM	watch on touchscreen	paper	other
	20	mean = 70.15	STEM	baseline	touchscreen devices	other
	20	mean = 70.15	STEM	baseline	physical objects	other
	21	mean = 70.15	STEM	baseline	paper	other
Wang et al., 2016	22	mean = 71.30	STEM	baseline	touchscreen devices	other
	21	mean = 70.80	STEM	baseline	physical objects	other
	22	mean = 69.30	STEM	baseline	paper	other
Willoughby et al., 2015	92	mean = 50.90	non-STEM	paper	oral	classroom
	33	mean = 50.90	non-STEM	baseline	oral	classroom
Xie and Zhou, in preparation	32	mean = 68.08	STEM	watch on touchscreen	touchscreen devices	other
	31	mean = 68.08	STEM	watch on touchscreen	paper	other
	16	mean = 68.08	STEM	baseline	touchscreen devices	other
	15	mean = 68.08	STEM	baseline	paper	other
Zipke, 2017 Exp.1	25	mean = 54.12	non-STEM	paper	oral	classroom
	25	mean = 54.12	non-STEM	paper	paper	classroom

### Overview of Present Study

Based on the detailed exposition of pedagogic effect of touchscreens mentioned above, an obvious and crucial issue concerns that the generality of touchscreen effect on young children's learning is an open question. These inconsistencies were the impetus for our meta-analytic investigation. Specially, this meta-analysis was conducted to address the following two questions:

RQ1: Is learning by physically touching a screen effective for increasing young children's learning performance?
RQ2: Is there any potential moderators of the effect of touchscreens on young children's learning?

Before widely introducing touchscreen devices to young children's learning at home or in preschool, it is of value to evaluate the overall effect of touchscreen learning. Thus, the most important question we addressed was whether or not young children would benefit from learning via touchscreen devices featured by physical interactivity. According to the potential role of physical experience in cognitive processing, we hypothesized that the learning outcome would be better in touchscreen condition compared to non-touchscreen condition.

In consideration of the mixed findings on the effect of touchscreens on child learning, one might argue that it is the moderators of this effect that counts. However, because this moderator-related question has not been attached enough importance, in most cases there is no theoretical rationale or sufficient empirical evidence to justify hypotheses about moderators. On an exploratory basis, we examined the following moderators (a) age, (b) learning material domain (STEM vs. non-STEM), (c) comparison condition (baseline vs. traditional classroom teaching vs. mouse-based computers vs. paper vs. physical objects vs. watch on touchscreen), (d) test media (touchscreen devices vs. paper vs. physical objects vs. oral), and (e) experimental environment (classroom vs. laboratory vs. other). We chose these variables as potential moderators because (1) when considering relevant empirical research as a whole, these variables were either continuous or able to be divided into different subgroups, which is necessary for moderator analyses, and (2) they were usually regarded as important potential moderators by researchers in the field of learning and instruction (e.g., Berney and Bétrancourt, 2016; Fiorella and Zhang, 2018; Xie et al., 2018). Since it was difficult to provide a theoretical frame for these potential moderators and to make predictions about the moderators' roles in the effect of touchscreens on young children's learning, moderation analyses in the present study were treated as exploratory, rather than theory-based. Even so, these potential moderators should prove of interest to touchscreen learning researchers.

## Methods

### Literature Search

To identify relevant studies on the effect of touchscreens on young children's learning, a systematic literature search was conducted by searching the electronic databases PsycINFO, Educational Resources Information Center (ERIC), Science Direct, ACM Digital library, IEEE Digital Library, and ProQuest. The search keywords were “touchscreen,” “tablet,” “iPad,” “mobile device,” and “educational apps” with different combinations of “learning,” “education,” “teaching,” “instruction,” and “educational performance.” All searches were conducted through first screening of abstracts and subsequent examination of full texts where appropriate. The reference sections of included articles were also subjected to forward and backward searches for other relevant articles. Some scholars (though very limited) in the field were personally contacted and asked to provide any other relevant unpublished work. Search engines such as Google Scholar and the reference lists of identified articles were also used. The literature search encompassed articles published up to July 2018.

### Study Selection

This meta-analysis, based on (quasi-)experimental or pretest-posttest designed research, mainly focused on the question of whether young children before elementary school who learned by touching a screen (touchscreen condition) outperformed those who learned without touching a screen (non-touchscreen condition) on learning performance tests. Given this goal, the studies were included for analysis if they met all of these criteria: (a) they were based on an experimental, a quasi-experimental, or a pretest-posttest design with empirical data; (b) normal participants ranged in age from 0 to 5, or the mean age was under 6 years old (not including 6 years old); (c) both a learning phase and a test phase existed; (d) children in a touchscreen group were asked to physically manipulate the interface of the screen (e.g., with a finger) during the learning phase; (e) there was a non-touchscreen/comparison group (baseline or other learning methods) in which children had no access to touching; (f) they measured the learning outcome (e.g., recall, comprehend, or transfer performance) during the test phase; (g) sufficient quantitative data (e.g., means, standard deviations and *n*; *t*-test or *F*-test values) were reported to calculate the effect size; and (h) no repetitive data were used.

**Figure 1:**
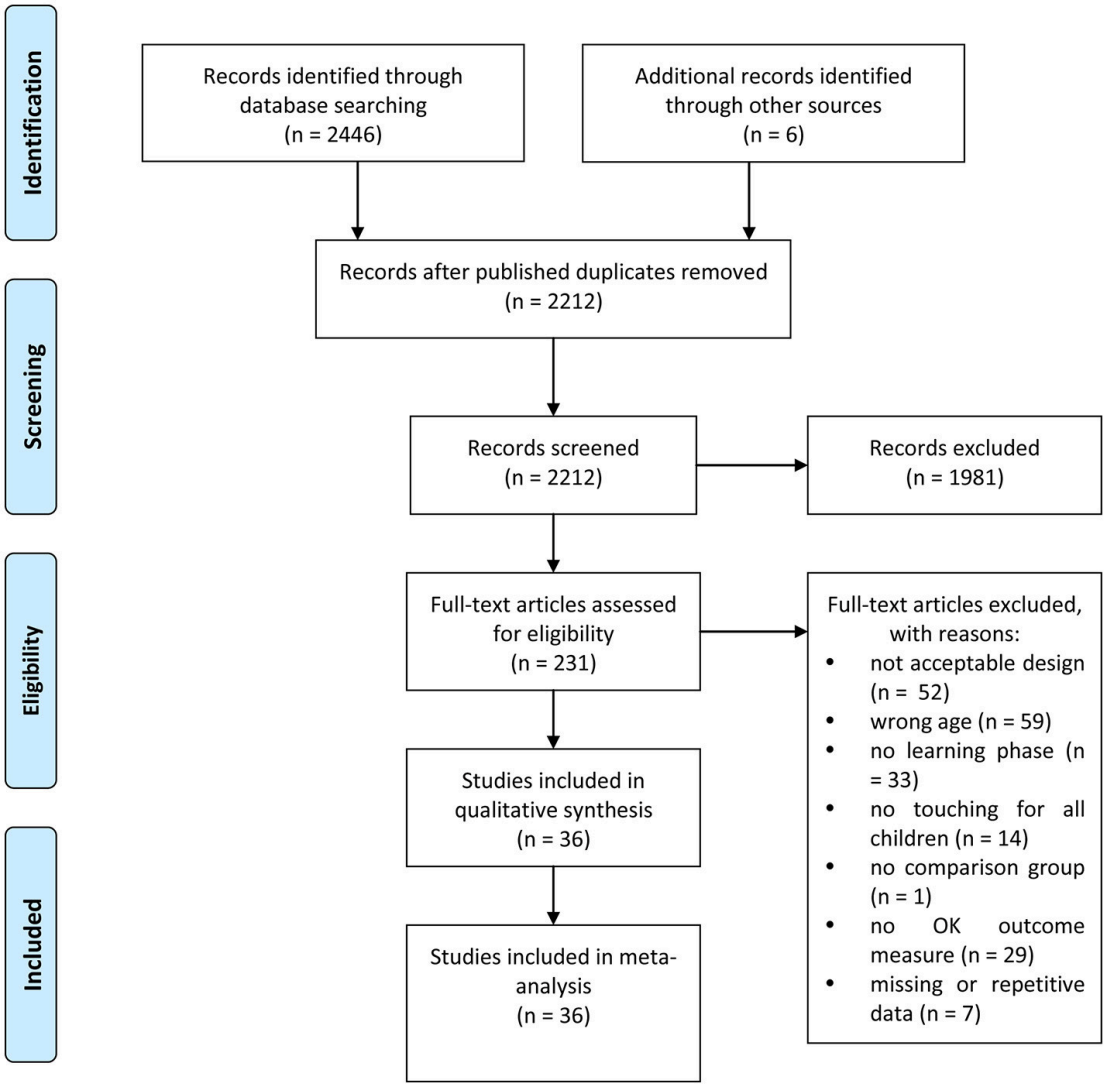
PRISMA flow diagram.

Accordingly, studies were excluded if: (a) they were description, opinion, review, case or correlational articles; (b) participants equal to or over 6 years old (e.g., primary school students, middle school students, adults) were recruited (e.g., Berkowitz et al., 2015; Volk et al., 2017); (c) the task was not related to learning (e.g., perception, sleep); (d) no children were allowed to physically touch the screen during the learning/demonstration phase (e.g., Zack and Barr, 2016; Zimmermann et al., 2017); (e) there was no comparison group (Dore et al., 2018); (f) only learning-irrelevant outcomes (e.g., motivational and emotional affordances or attitudes of touchscreen devices) were tested; (g) statistical data were insufficient; and (h) they used repetitive data (e.g., Papadakis et al., 2016). Figure 1 presents PRISMA flow diagram for the literature search, showing the number of studies identified, screened, found to be eligible, and finally included in the meta-analysis.

### Coding of Studies

Three types of information were collected from each study (see Table 1): basic information (authors, year of publication, sample size), quantitative information for the calculation of effect sizes, and characteristics related to the potential moderators (age, learning material domain, comparison group, test media, and experimental environment).

1. Age. Participants' mean age was coded. The unit was converted to month. For the studies investigating age difference of touchscreen learning, data were extracted and coded according to different age groups (e.g., Moyer-Packenham et al., 2015; Tarasuik et al., 2017). For example, Kirkorian et al. (2016) divided their sample into three age groups, namely young age children (23.5–27.5 months), middle age children (27.5–32.0 months), and old age children (32.0–36.0 months). Data of this study were respectively extracted and coded based on each age group^2^. This was done for guaranteeing age-related analysis.
2. Learning material domain. Research on touchscreen learning has used instructional materials from various domains, such as science, technology, engineering, mathematics (known collectively as STEM), or non-STEM domain. For example, some studies asked participants to learn measuring (Aladé et al., 2016), scientific trivia knowledge (Kwok et al., 2016), or how to tell time (Wang et al., 2016), etc. These kinds of studies were combined into a single category–STEM, to maximize the number of studies in this category. However, materials related to story comprehension (Piotrowski and Krcmar, 2017), language arts (Bebell and Pedulla, 2015), word learning (Russo-Johnson et al., 2017), or puzzle problem solving (Huber et al., 2016) were also used in some studies. This set of studies was combined and coded as non-STEM.
3. Comparison group. Among the included studies, the touchscreen group was usually compared with various groups, such as baseline, traditional classroom teaching, mouse-based computers, paper, physical objects, or watch on touchscreen. In (quasi-)experimental designed studies, if the touchscreen group was compared with a group in which participants were asked to complete a non-learning task (e.g., Aladé et al., 2016), then the comparison group was classified as baseline. In addition, if the post-test score of the touchscreen group was compared with its pre-test score (e.g., Wang et al., 2016), then it was also classified as baseline. The other comparison groups (i.e., traditional classroom teaching, mouse-based computers, paper, physical objects, and watch on touchscreen) were coded according to what the comparison actually was. For instance, Patchan and Puranik (2016) taught one group of preschool children to write letters by using iPad and the other group by paper. Thus, the comparison group of this study was naturally classified as paper. In addition, when several non-touchscreen conditions were compared with a touchscreen condition, the comparison groups were coded respectively. For example, Papadakis et al. (2018) simultaneously compared the effectiveness of touchscreen tablets (group 1), computers (group 2), and traditional classroom teaching (group 3) in early childhood students' understanding of numbers. When comparing group 1 with group 2, it was categorized as mouse-based computers. However, when comparing group 1 with group 3, it was categorized as traditional classroom teaching. It should be noted that comparison between learning by touching a screen and baseline reflects the effect of touchscreen itself, whereas comparison between learning by touching a screen and other learning methods (i.e., traditional classroom teaching, mouse-based computers, paper, physical objects, and watch on touchscreen) reflects the relative effect of touchscreens. Thus, analyzing this potential moderator contributes to making a comparison between the effect of touchscreen *per se* and its effect relative to other learning methods.
4. Test media. Participants in different studies were usually tested by various media, such as touchscreen devices, paper, physical objects, or oral test. Coding studies into these categories was based on what the test medium actually was. For example, in Piotrowski and Krcmar's (2017) work, all children's comprehension was measured via a paper-based multiple choice questionnaire. Therefore, the test medium of this study was naturally classified as paper. Likewise, when different kinds of test media were simultaneously analyzed in a single study, they were coded, respectively (e.g., Wang et al., 2016).
5. Experimental environment. The included experiments were usually conducted in different environments, such as classroom, laboratory. Coding studies into these categories was based on what the exact environment was. For example, Russo-Johnson et al.'s (2017) data were collected in a lab room, and it was coded as laboratory. Some intervention studies collected data from children's classrooms and thus they were classified as classroom (e.g., Oakley et al., 2018). However, for the purpose of convenience, some researchers collected part of their data in the classroom and/or laboratory (Schroeder and Kirkorian, 2016), a child care center (Piotrowski and Krcmar, 2017), or even an empty dancing room of the preschool (Wang et al., 2016). The experimental environment of these studies was classified as other.

The included studies were double-coded and reliability estimates calculated using kappa ranged from 0.81 to 0.92, which are considered to be acceptable (Mchugh, 2012).

### Calculation of Effect Sizes

Data were analyzed using the Comprehensive Meta-Analysis (CMA) 2.0 software (https://www.meta-analysis.com/). Effect sizes were weighted using the reciprocal of their variances so that effect sizes based on studies with larger sample sizes were more heavily weighted in the analysis. The random-effects model was used for analyses because studies included in the meta-analysis differed on a number of variables (e.g., characteristics of participants, research design and procedures), conforming to the assumption of the random-effects model that the true effect sizes are not exactly the same in all studies (Borenstein et al., 2009).

Cohen's *d* was selected as the standardized estimate of effect size (Cohen, 1988). Specifically, Cohen's *d* was calculated as the mean score difference in learning outcome between an experimental group and a comparison group or between a posttest and a pretest. When a study reported multiple conditions related to the moderators we wanted to examine, we separately calculated the subgroup effect sizes in order to test for moderation effects. For example, Aladé et al. (2016) used a between-subjects design with three experimental conditions (i.e., play an interactive game, view a video recorded version of the game, and play a similar but learning-irrelevant game); for this study, two effect sizes were computed for the moderator “comparison group”: one was calculated by contrasting the first condition with the second condition and was coded as “watch on touchscreen,” the other one was calculated by contrasting the first condition with the third condition and was coded as “baseline.”

The generated effect sizes were then averaged to obtain an overall average effect size point estimate for quantifying the central tendency among the effect sizes. A forest plot with 95% confidence interval (95% CI) for each effect size, organized by dependent variable, was created to detect patterns in the magnitude of the individual effect sizes. For Cohen's *d*, the direction of the effect size was positive if participants' learning outcome of the experimental group or at posttest was of greater magnitude than that of the comparison group or at pretest. The magnitude of an effect size was interpreted using Cohen's (1992) standards of small (*d* = ± 0.20), moderate (*d* = ± 0.50), and large (*d* = ± 0.80). Additionally, we reported the 80% credibility intervals (80% CV) of the corrected population effect size. If a credibility interval is large and includes zero, it indicates that there is considerable variation across studies and moderators are likely operating (Whitener, 1990).

### Homogeneity Test

Two tests were used to determine if there was a significant degree of heterogeneity in the data. Significant heterogeneity suggests that the random-effects model is reasonable and that there is a call for tests of moderation. The homogeneity statistic *Q*, along with its *p*-value, was used to test whether there was significant variance within the set of effect sizes for learning outcome. A related statistic, *I*^2^, was used to estimate the percentage of total variance that was due to true between-study heterogeneity rather than random error. *I*^2^ values of around 25, 50, and 75% are generally interpreted to indicate low, medium, and high heterogeneity, respectively (Higgins et al., 2003).

### Evaluation of Publication Bias

Publication bias is considered to emerge in meta-analyses if there are systematic errors between articles that ought to be included and those actually included (Borenstein et al., 2009). In the present work, we first calculated the fail-safe *N* (*N*_fs_) to detect potential publication bias (Rosenthal, 1979). The *N*_fs_ shows how many (probably unpublished) studies with null effects would be needed to turn a significant effect size into a nonsignificant one. A large *N*_fs_ (larger than 5*k* + 10, with *k* being the number of effect sizes in the meta-analysis) means it is unlikely that there was publication bias (Rosenthal, 1979). Second, we performed Egger's linear regression test (Egger et al., 1997), with the standard normal deviate of each study as the dependent variable and the estimate's precision in each study as the independent variable. The smaller the intercept's deviation from zero the less pronounced the publication bias.

## Results

### Descriptive Analysis

**Figure 2:**
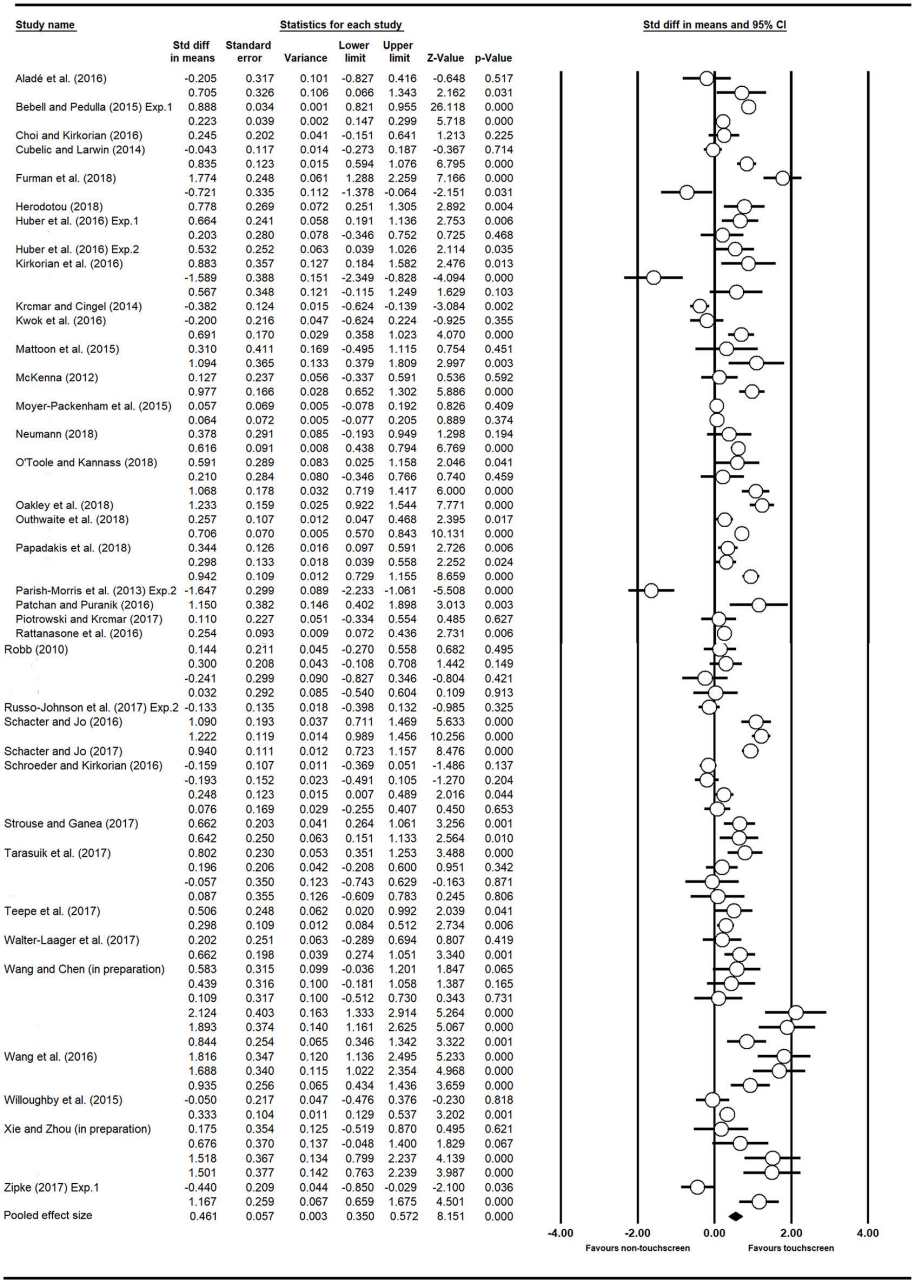
The forest plot of individual effect sizes.

A total of 36 empirical articles that met the inclusion criteria were finally included and analyzed. An overview of the 36 articles with basic information and coded moderators is presented in Table 1. Most of the articles were published after 2016 (75.0%) and were obtained from journals (91.7%). Across the 36 studies, 79 effect sizes were computed, involving 4,206 participants. There were 65 out of 79 positive effect sizes (82.3%). The mean age ranged from 21.33 months to 71.30 months. Figure 2 presents the forest plot with the point estimate of each effect size with a 95% confidence interval.

### Overall Analyses

Table 2 presents the results regarding the effect of touchscreen devices on young children's learning outcome. The meta-analysis revealed that the overall pooled effect size was statistically significant and medium in magnitude (*d* = 0.46, *p* < 0.001). Thus, better learning outcome was found in touchscreen condition compared to non-touchscreen condition, indicating that using touchscreen devices promoted young children's learning performance.

**Table 2:** The main effect of using touchscreen devices on young children's learning outcome.

**Dependent variable**	***N***	***k***	**Effect size**					**Homogeneity test**		
			**Cohen's *d***	***p***	**95% CI**	***z***	**80% CV**	***Q***	***p***	***I*^**2**^**
Learning outcome	4,206	79	0.46^***^	< 0.001	[0.35, 0.57]	8.15	[−0.19, 1.11]	866.20	< 0.001	91.00

*N, total number of participants; k, number of effect sizes; CI, confidence interval; CV, credibility interval*;

*** *p < 0.001*.

As shown in Table 2, the large credibility interval (80% CV = [−0.19, 1.11]) suggested that moderating variables were operating. In addition, the homogeneity test showed that effect sizes varied significantly across studies (*p* < 0.001), with a very high heterogeneity due to variance across studies (*I*^2^ > 90). These results warranted tests of moderation to identify sources of this heterogeneity.

### Moderator Analyses

**Figure 3:**
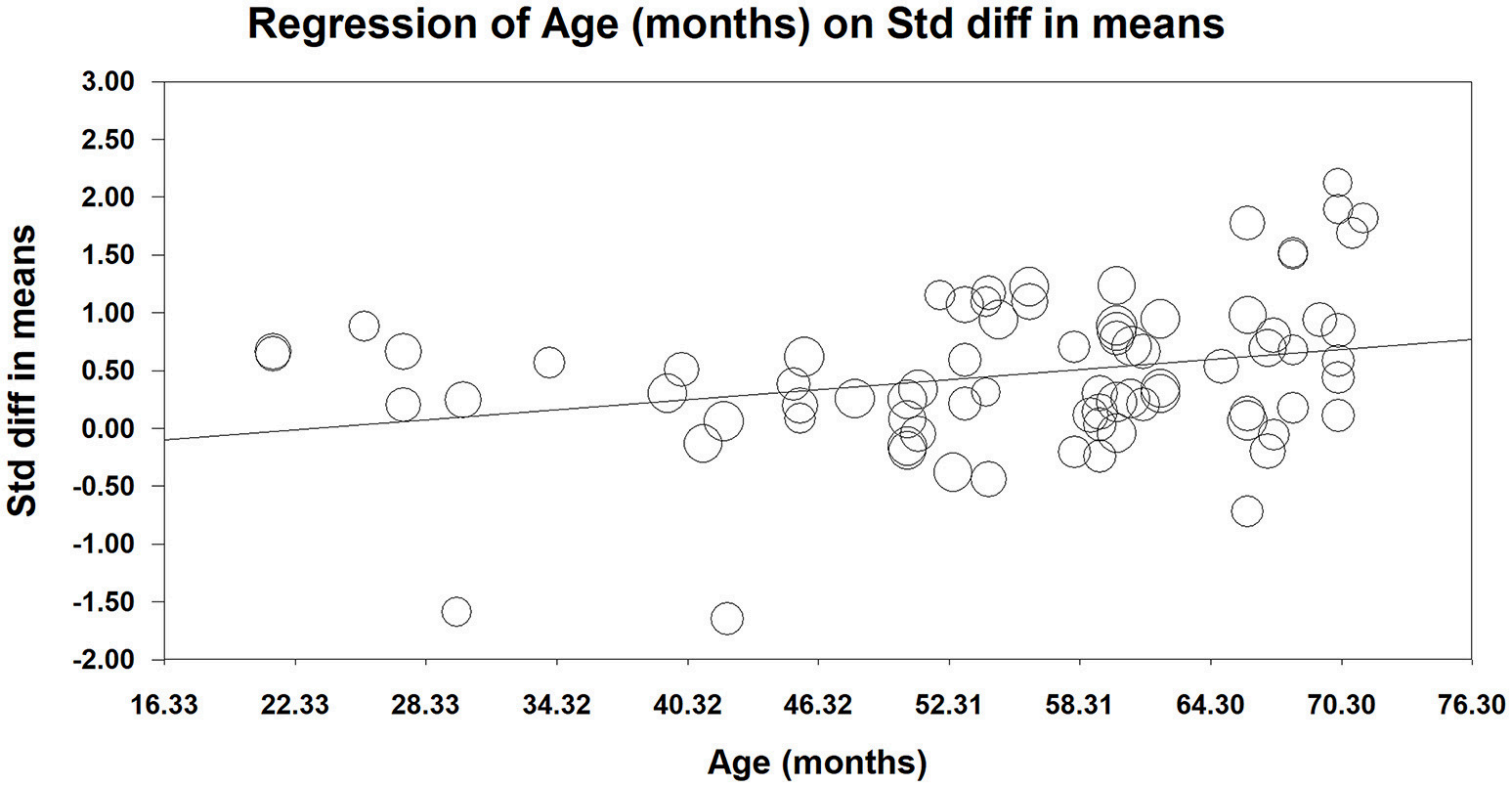
Age-related meta-regression analysis (The size of the circle is proportional to study weight).

Figure 3 and Table 3 present the results of the moderation analyses. Because age is a continuous variable, a meta-regression analysis was conducted for this potential moderator (see Figure 3). The result revealed that the effect of using touchscreen devices on young children's learning performance was significantly moderated by age (β = 0.015, 95% CI = [0.006, 0.023], *p* = 0.0013). The effect size increased with age.

**Table 3:** Moderator analyses on young children's touchscreen learning.

**Variables**	***N***	***k***	**Effect size**					**Homogeneity test between subgroups**	
			**Cohen's *d***	***p***	**95% CI**	***z***	**80% CV**	***Q*_**B**_ (*df*)**	***p***
Learning material domain								8.23(1)	0.004
STEM	1,907	37	0.65^***^	< 0.001	[0.47, 0.82]	7.20	[−0.05, 1.35]		
Non-STEM	2,299	42	0.31^***^	< 0.001	[0.16, 0.46]	4.04	[−0.32, 0.94]		
Comparison group								39.47(5)	< 0.001
Baseline	1,596	34	0.77^***^	< 0.001	[0.62, 0.93]	9.99	[0.19, 1.36]		
Traditional classroom teaching	2,215	12	0.46^***^	< 0.001	[0.20, 0.71]	3.53	[0.01, 0.90]		
Mouse-based computers	256	1	0.34^**^	0.006	[0.10, 0.59]	2.73	[0.18, 0.51]		
Paper	658	13	0.11	0.537	[−0.24, 0.46]	0.62	[−0.71, 0.93]		
Physical objects	99	3	0.10	0.600	[−0.27, 0.46]	0.52	[−0.31, 0.51]		
Watch on touchscreen	754	16	0.07	0.502	[−0.13, 0.27]	0.67	[−0.45, 0.59]		
Test media								6.62(3)	0.085
Touchscreen devices	977	13	0.73^***^	< 0.001	[0.43, 1.04]	4.69	[0.01, 1.45]		
Paper	2,567	39	0.48^***^	< 0.001	[0.34, 0.62]	6.77	[−0.09, 1.05]		
Physical objects	339	15	0.41^*^	0.015	[0.08, 0.73]	2.42	[−0.43, 1.24]		
Oral	368	9	0.06	0.765	[−0.35, 0.48]	0.30	[−0.75, 0.88]		
Experimental environment								10.24(2)	0.006
Classroom	2,659	27	0.55^***^	< 0.001	[0.38, 0.73]	6.12	[−0.05, 1.15]		
Laboratory	675	21	0.20^*^	0.027	[0.02, 0.37]	2.21	[−0.33, 0.73]		
Other	872	31	0.55^***^	< 0.001	[0.37, 0.74]	5.88	[−0.12, 1.22]		

* *p < 0.05*;

** *p < 0.01*;

*** *p < 0.001*.

Because the remaining potential moderators are categorical variables, subgroup analyses were conducted for them (see Table 3). Regarding learning material domain, the moderating effect was found to be significant (*Q*_B_ = 8.23, *p* = 0.004). Comparing to non-STEM knowledge, young children benefited more from touchscreens when learning STEM knowledge.

Regarding comparison group, the result showed a significant moderating effect (*Q*_B_ = 39.47, *p* < 0.001). Young children benefited more from learning with touchscreens when it was compared to baseline group than when it was compared to learning with traditional classroom teaching (*Q*_B_ = 4.46, *p* = 0.035), mouse-based computers (*Q*_B_ = 8.48, *p* = 0.004), paper (*Q*_B_ = 11.79, *p* = 0.001), physical objects (*Q*_B_ = 11.27, *p* = 0.001), and watch on touchscreen (*Q*_B_ = 30.68, *p* < 0.001).

Regarding test media, the between-level difference was not statistically significant (*Q*_B_ = 6.62, *p* = 0.085).

Finally, regarding experimental environment, the result showed a significant moderating effect (*Q*_B_ = 10.24, *p* = 0.006). Touchscreen learning in classrooms was more beneficial to young children's performance than in laboratories (*Q*_B_ = 7.72, *p* = 0.005).

### Publication Bias Analysis

The calculation of Rosenthal's *N*_fs_ indicated that it would take 1,255 studies with non-significant findings on task performance before the cumulative effect in the meta-analysis would no longer be statistically significant. This is considered a robust effect (Rosenthal, 1979). Egger's linear regression test also showed that publication bias was an unlikely influence on the findings of the present meta-analysis (intercept = −0.28, *p* = 0.648).

## Discussion

Although there have been many empirical studies examining the effect of using touchscreen devices on young children's learning outcome, the results have been mixed. The current meta-analysis is one more successful attempt to provide an empirical investigation of the overall effect of touchscreen learning on young children's performance and potential moderators of this effect.

The most important test in the present study addressed whether using touchscreens could facilitate young children's learning outcome. The overall analysis provided a positive answer to this question. Young children who learned with touchscreen devices indeed performed better than those who had no access to touching (*d* = 0.46), which is in line with our hypothesis as well as numerous studies (e.g., Patchan and Puranik, 2016; Schacter and Jo, 2016; Wang et al., 2016; Strouse and Ganea, 2017). Thus, the current study shows empirical evidence on the superiority of touchscreens featured with physical interactivity in samples of young children from birth to 5 years of age.

Interestingly, the exploratory moderator analyses showed that age, learning material domain, comparison group, and experimental environment significantly moderated the effect of touchscreen devices on young children's learning outcome. First, the effect size of touchscreen learning vs. non-touchscreen learning increased with children's age. One might argue that it is the ability of imagine/mental manipulation that counts. A line of previous evidence indicates that there is a close relationship between cognitive processing and mental manipulation (González and Kolers, 1982; Shepard and Cooper, 1982; Driskell et al., 1994; Kosslyn et al., 2001), and the role of children's imagination during learning processes was emphasized to some extent (Egan, 1994; Glenberg et al., 2004). For young children like pre-schoolers, their ability of imagination develops with age (Piaget, 1945; Diachenko, 2011). Thus, presumably, learning might be improved for older children (rather than younger children) with the combined help of physical manipulation on a touchscreen and their relatively good capability of imagination. Of course, further direct work is needed to determine whether it is the coordinated role of touchscreen and imagination that counts in older children's learning performance. Second, young children benefited more from touchscreens when learning STEM knowledge compared to non-STEM knowledge. This might be because STEM-related concepts are more easily comprehended when they are learned via physical experience, and touchscreens provide more of a “real-life” experience which is important for STEM learning (Han and Black, 2011; Aladé et al., 2016). Third, young children benefited more from touchscreen learning when comparing touchscreen with baseline than when comparing it with other non-touchscreen learning methods. The comparison between touchscreen and baseline reflects the effect of touchscreen *per se* (Wang et al., 2016), whereas the comparison between touchscreen learning and other learning methods (e.g., paper learning) reflects the relative role of touchscreen. Thus, the significant moderating effect of comparison group indicates that the effect of touchscreen itself tends to be stronger than its relative effect. Finally, touchscreen learning in classrooms was found to be more beneficial to young children than in laboratories, which could be explained by the fact that the nature of learning in a laboratory environment changes because of various factors (e.g., test expectation), thus discounting the touchscreen learning effect. It should be pointed out that (1) because the above interpretations of moderating effect results somewhat deviated from the notion of physical experience, much attention should be paid to more powerful theoretical explanations in future research related to these moderators and, (2) because the number of included studies was small to a certain extent and there were very few studies represented in some subgroups (e.g., mouse-based computers, oral), the results might be susceptible to potential false positives and must be treated with some caution.

Our results shed light on the role of touchscreen-related physical experience in early childhood education and hold promise for using touchscreens with physical interactivity function to scaffold young children's learning in either formal or informal educational settings. With the help of touchscreens, the physical experience can be either long-term accumulation of experience or salient short-term experience. Either way, parents, teachers or educational practitioners should, at least partly, provide instructional support to touchscreen learning. However, it should be acknowledged that touchscreens are not suggested as educational intervention techniques in any condition or at any age point because the current study can not respond to the question whether using touchscreen devices has underlying negative influence on other aspects (e.g., sleep quality, the ability of deferred gratification).

There are at least several limitations that should be acknowledged. First, in this study we did not examine whether there is a touchscreen learning effect in samples of children over 5 years old or adults, a question that needs to be discussed in future research. Second, we could not distinguish the different effects of touchscreens under different levels of interactivity. The interface of touchscreen devices affords both high level of interactivity (e.g., rotating an object by dragging or zooming) and low level of interactivity (e.g., tapping some specific objects or pause/play buttons) (Pedra et al., 2015), which might show different roles in touchscreen learning. Third, only learning outcome was examined in this study. Perhaps other dependent variables (e.g., related to cognitive development, attitudes on touchscreens) would show additional unexpected but vital results. Fourth, caregiver-child interaction was not investigated in the present study because there were only a very small number of included studies investigating the effect of caregiver-child interaction on young children's learning performance. Thus, another interesting direction for future research would be to investigate the different effects of touchscreens when a caregiver was present or absent during young children's learning. Finally, in order to test for moderation effects, some of the subgroup effect sizes were separately calculated even though they were not independent of each other. This limitation might be addressed as the number of empirical studies increases.

## Author Contributions

HX and ZZ developed the study concept and design. HX, JP, MQ, and XH were involved in data collection. HX and FT were involved in statistical analyses. All authors contributed to writing and editing the manuscript. All authors approved the final version of the manuscript for submission.

### Conflict of Interest Statement

The authors declare that the research was conducted in the absence of any commercial or financial relationships that could be construed as a potential conflict of interest. The reviewer, RL, and handling Editor declared their shared affiliation.

## Footnotes

^1^Learning performance (or learning outcome) refers to the observed knowledge or skill acquisition. It usually can be reflected as the extent to which students recall, comprehend, or transfer what they have learned from an instructional task (Mayer, 2009).

^2^In Study 2 of Parish-Morris et al.'s (2013) work, five-year-old children in both touchscreen and non-touchscreen conditions demonstrated ceiling effects in all tasks and only the comprehension data in 3-year-old group sample were provided. For this reason, we only extracted and coded the 3-year-old data with respect to this work.
